# Patient Views on Advance Care Planning in Cirrhosis: A Qualitative Analysis

**DOI:** 10.1155/2018/4040518

**Published:** 2018-07-18

**Authors:** Michelle Carbonneau, Tracy Davyduke, Jude Spiers, Amanda Brisebois, Kathleen Ismond, Puneeta Tandon

**Affiliations:** ^1^Alberta Health Services, 8440 112 Street, Edmonton, AB, Canada T6G 2B7; ^2^Cirrhosis Care Clinic, 8540 112 Street, Zeidler Ledcor Centre, Edmonton, AB, Canada T6G 2X8; ^3^University of Alberta, Faculty of Nursing, 3-141 Edmonton Clinic Health Academy (ECHA) 11405 87 Avenue, Edmonton, AB, Canada T6G 1C9; ^4^University of Alberta, Faculty of Medicine, 2J2.00 Walter C Mackenzie Health Sciences Centre, 8440 112 St., Edmonton, AB, Canada T6G 2R7; ^5^University of Alberta, Department of Oncology, Division of Palliative Care, 2J2.00 Walter C Mackenzie Health Sciences Centre, 8440 112 St., Edmonton, AB, Canada T6G 2R7

## Abstract

**Aim:**

To investigate patient experiences and perceptions of advance care planning (ACP) process in cirrhosis.

**Methods:**

Purposive sampling was used to identify and recruit participants (N = 17) from discrete patient groups: compensated with no prior decompensation, decompensated and not yet listed for transplant, transplant wait listed, medical contraindications for transplant, and low socioeconomic status. Review and discussion of local ACP videos, documents, and experiences with ACP occurred in two individual interviews and four focus groups. Data were analyzed using inductive content analysis including iterative processes of open coding, categorization, and abstraction.

**Results:**

Three overarching categories emerged: (1) lack of understanding about disease trajectories and ACP processes, (2) roles of alternate decision makers, and (3) preferences for receiving ACP information. Most patients desired advanced care-planning conversations before the onset of decompensation (specifically hepatic encephalopathy) with a care provider with whom they had a trusting, preexisting relationship. Involvement of the alternate decision makers was of critical importance to participants, as was the use of direct, easy to understand patient education tools that address practical issues.

**Conclusion:**

Our findings support the need for early advance care planning in the outpatient setting. Outpatient clinicians may play a key role in facilitating these discussions.

## 1. Introduction

As cirrhosis progresses, patients experience frequent and sudden deteriorations in health that result in repeated hospitalizations and mortality [1]. Although time to death varies from 1 to 20 years, individuals usually progress from compensated to decompensated cirrhosis, with functional decline and complications [2]. These patients have a high symptom burden resulting in significantly impaired health related quality of life [3, 4]. Liver transplantation, the only definitive treatment option, is available to less than 10% of affected patients and approximately 20% of patients on the liver transplant wait list die or are removed from the list for being too sick [5, 6].

Palliative care minimizes symptom burden without modifying the disease trajectory [7–9]. An integral characteristic of palliative care is preparing the patient and their family with advance care planning (ACP) and end of life (EOL) options [10]. ACP is often not addressed in this population until late in the disease trajectory with one study reporting that of all the patients who had been denied liver transplant only 28% had a Goals of Care Designation (GCD) documented in their medical charts [11, 12].

Clinician barriers to initiation of ACP conversations include concern about upsetting patients, patient cognitive capacity, lack of training or discussion aids, time limitations, and, in some situations, a focus of care on liver transplantation [7–9, 11–15]. Identifying the most appropriate time to initiate ACP/GCD discussions is difficult, as patient preferences may vary. Decisions made when one is feeling well may be different than those made when being acutely unwell [16]. Patient barriers to ACP include lack of understanding about disease trajectory, social factors, and discomfort with planning for EOL [14, 17].

### 1.1. Standardized ACP and GCD

In 2014, Alberta Health Services (AHS), the sole health authority for the province of Alberta, standardized ACP and GCD documentation and practices [18]. GCDs are formal clinician orders resulting from conversations and determination of preferences between the clinician and patient [19]. GCD details are divided into categories (Table 1) to describe the general focus of care, limits to interventions, and location of care [20]. The decisions are recorded on a standard form which is retained by the patient and brought to outpatient appointments or hospital admissions, to be reviewed or altered as required [20]. GCDs are most often completed in the hospital setting; however, it has been reported that less than 30% of hospitalized patients are aware they had a GCD [21].

The purpose of this study was to explore perspectives and experiences of patients regarding the processes of ACP/GCD in the setting of cirrhosis.

## 2. Materials and Methods

This study employed qualitative description as described by Sandelowski [22], with a goal to ensure findings reflected the participants' views in terms that remain close to the data. Interviews and focus groups allowed us to investigate patients' experiences and perceptions with ACP/GCD. We also aimed to understand how the AHS ACP/GCD resource materials, including videos and pamphlets, were perceived and if or how they had been used in the participants' own experiences. Activity oriented focus groups are particularly useful in gathering reflective data and to encourage participants to answer questions in more active ways [23]. Guiding questions were designed by the investigative team based on the current literature, and for the purposes of understanding and determining what values and information were important to participants. The study received ethics approval from the University of Alberta and was part of a larger quality improvement initiative aimed at understanding and improving ACP processes in cirrhosis.

### 2.1. Participants

Participants were recruited from the Cirrhosis Care Clinic at the University of Alberta Hospital. Purposive maximum variation sampling [22] was used to identify and invite a minimum of three study participants from each of the following discrete patient groups: compensated with no prior decompensation (N = 3), decompensated, not yet listed for transplant (N = 4), transplant wait listed (N = 3), medical contraindications for transplant (N = 3), and low socioeconomic status (SES) (N = 4). The low SES participants all had decompensated cirrhosis and were included in a discrete group for the purpose of understanding perspectives unique to this population. Potential participants were identified via chart review and were recruited via personal phone call by the primary investigator (PI) who is also a Nurse Practitioner in the Cirrhosis Care Clinic. Two people declined to participate; one person consented but was hospitalized the day of the focus group and could not participate. Two participants requested one on one interviews as opposed to participating in the focus groups, one of which requested to include a spouse.

### 2.2. Data Collection

The PI (MC), an experienced Nurse Practitioner, collected all data. The PI had professional contact with some participants but had not been involved in ACP/GCD discussions with any participant. Data collection occurred in April-August 2016, in two individual interviews (one including a spouse) and four focus groups containing 3–5 participants. All interviews and focus group meetings took place in a private meeting room within the clinic building. The focus groups were kept small due to the sensitive nature of the topic, to encourage comfort and interaction among the participants. Written consent was obtained before each interview or focus group. Interviews/focus groups were semistructured in format and consisted of two parts. In the first ten minutes, participants viewed the AHS ACP and GCD education videos [24, 25] that are used to help patients/alternate decision makers understand the concepts and start the process of thinking about their values, preferences, and options. Participants then viewed and discussed the AHS standardized ACP tracking record and GCD documentation form. Personal Directive (PD) forms were also presented for discussion. It was necessary to refresh participants' memories of these resources and documents as they may have been exposed to ACP/GCD discussions in multiple arenas including the community, hospital, intensive care, and emergency department visits over significant periods of time. Reviewing these resources allowed the participants to reflect on the messages, presentation, and language in the materials and acted as a prompt to recall their own experiences. A series of open-ended questions followed by probing questions were used to explore participants' experiences, beliefs, and attitudes about advance care planning (Box 1). Participants were also encouraged to raise their own questions and concerns beyond the topics raised through the interviewer's prompts. No restrictions were placed on the length of the interviews or focus groups (which lasted 60-90min) in order to allow participants time to freely express their individual perspectives.

### 2.3. Data Analysis

Data were transcribed and analysis was managed using NVivo 10©. Analysis was performed using inductive content analysis procedures including iterative processes of open coding, categorization, and abstraction [26]. Each transcript was repeatedly reviewed and independently coded by three researchers (MC, TD, and JS). Team discussions ensured consensus about interpretation and coding and the collapsing of codes into subcategories, generic categories, and main categories [26]. Analysis of focus group transcripts also included latent content coding of nonverbal behaviors that informed interpretation of the verbal information. The final analysis was reviewed by two additional team members (AB and PT). Any differences in interpretation were resolved by consensus in team discussions.

## 3. Results

Demographic and clinical characteristics of participants are presented in Tables 2 and 3. Each participant was given a study identification number ACP 1-17, based chronologically on the date consent was signed.

Uncertainty about their disease trajectory was a major factor in participants' levels of readiness to engage in ACP discussions and decision-making. Many participants had completed legal documents such as wills and advanced directives. Few, however, appeared to understand the role and values underlying the GCD process or forms even if they did, in fact, possess a signed GCD form. Participants broadly realized the GCD process was intended to ensure their wishes were followed and to reduce decisional burden on families, although there was some indication that the GCD was seen more of a bureaucratic measure designed to safeguard the healthcare system against liability. Overall, participants indicated ACP/GCD discussions that occurred in acute care situations when they were experiencing an exacerbation of their condition were dispreferred. Three overarching categories emerged: (1) lack of understanding about disease trajectories and ACP processes, (2) role of family/caregivers in care and ACP decision-making, and (3) preferences for receiving ACP information.

### 3.1. Lack of Understanding about Disease Trajectories and ACP Processes

Participants expressed an overall lack of understanding regarding cirrhosis trajectories and the role of ACP processes. Participants with clinical decompensation wanted clinicians to clearly explain key stages in the progression of cirrhosis, treatment options, and outcomes to better manage their expectations:

I would've liked to have known right from the beginning, what levels are going to happen next … If you predict when the end is near, I want to know…. You… know you have the disease and the hope is transplant later on, but [if] you know they're not going to do that … what are the other options to manage symptoms? (Medical Contraindication to Transplant, ACP16)

Most participants stated they were unsure about the differences between legal wills, PDs, and the GCD. Less than half of the participants had a PD and none of the low SES participants had a PD in place. Those waiting for transplant either had a PD prepared or had talked about it, as had most participants from the medical contraindication to transplant group. For these participants, working through formal ACP processes reflected their disease stage, in that wills were completed soon after diagnosis, and PDs were completed as they experienced exacerbations or were faced with transplant or the decision that transplant was not an option. Those with wills and PDs regarded them as necessary decisions that can then be “*put it aside, like an insurance you hope to never use*”* (Compensated, ACP1)*.

Only one participant accurately articulated the intent and function of the GCD form. Interestingly, the 13 previously hospitalized participants had a GCD but were unaware of its purpose. A typical comment was, “I have one of those [GCD form] at home but I don't know what it's for.” (Low SES, ACP5). Participants who had experienced decompensation typically felt that GCDs had been determined in emergency situations:

I was really close to dying but I pulled out of it. They gave the green sleeve [GCD form] to me at that time. Maybe it was because they thought I was going to die. (Low SES, ACP4)

Few participants clearly recalled the ACP discussions that had led to their GCD, because the GCD had been completed during a period of acute illness. While looking at a GCD sample document, one participant vaguely remembered, but inaccurately identified it:

This looks familiar. I think I may have got one in hospital, on one of my trips to the hospital. Personal directive, right? (Decompensated, not yet listed for transplant, ACP15)

A participant on the transplant wait list recounted that it was only after he had recovered that the GCD was explained:

I did actually get mine (GCD form) in the hospital and the nurse came and said ‘you probably got this but they didn't explain what it is', and then she went through it with me...looks like it's written by an insurance company. (Transplant Wait Listed, ACP9)

Participants were often unclear about whether the PD or the GCD held precedence. One participant from the Low SES group, who had been hospitalized numerous times, had three different GCDs. Although some knew they had a GCD, few recalled its intent.

### 3.2. Role of Alternate Decision Makers

Most participants had an alternate decision maker (N = 14/17), and most often named a spouse. They reported a significant reliance on their families during periods of symptom exacerbation:

I'm lucky that my husband was able to help, but there are times when he's found it frustrating. I'll be fine one day and then I'm gone- I can't even speak [referring to encephalopathy]. (Contraindication to Transplant, ACP16)

Most described their alternate decision makers as having a significant role in their health decision-making as this participant described:

My wife, she looks after me and she knows a lot about all this stuff...what she says, they listen, so I don't have to say very much. I'm quiet. (Transplant Wait Listed, ACP10)

Many described a personal preference for their families not to see them in an intensive care setting at EOL and saw the ACP as critical to reducing the decision-making burden on family. The existence of PDs created when the person was healthier was viewed as a way to ensure their wishes would prevail should family members disagree with their wishes:

Because when the time comes, then the fight starts. Maybe one of your children doesn't want to let go so when you have a personal directive that child stops, and you get to make those decisions when you're in a healthier place. (Contraindication to Transplant, ACP17)

However, when participants felt well they appeared less motivated to initiate ACP discussions with family:

I've been meaning to do this for forever. It's one of those things you think, yeah that's a good idea… we've talked about my disease but I look healthy … family look at you and think you look okay. You've never thought, sat down and said … this is what should happen … (Compensated, ACP2)

Many participants spoke about difficulties in discussing EOL issues with their families. They expressed the desire for information and guidance on how to have these discussions:

I try to [have these discussions] with my children and they say ‘you're not dying'. They don't want to think about it. It becomes very hard. It is emotional for my wife too, but she understands more. (Contraindication to Transplant, ACP17)

Participants in the low SES group expressed concern over lack of family support and substitute decision makers:

My daughter doesn't talk to me. I have no family, no nothing. I don't really have any friends, so what am I supposed to do? (Low SES, ACP4)

They were concerned about the possibility of family making poor decisions at EOL, as one person suggested, “*What if they (family) say leave him in pain forever, he owes us?” (Low SES, ACP5).*

### 3.3. Preferences for Receiving ACP Information

The uncertainty of determining a prognosis was a major issue for most participants. ACP conversations had occurred as a process that reflected acute changes in their illness trajectory. However, when asked when ACP/GCD conversations should occur, all agreed that discussions and GCD decisions should happen outside of hospital and not during periods when they were acutely ill:

We all are hoping we're going to be terrifically fine, but you don't know that's going to happen and that's why I think earlier the better because later you might not be ‘all there' [referring to encephalopathy], and that's very important. (Low SES, ACP6)

Some participants worried that situations when symptoms were worse would negatively affect their ability to pay attention and make decisions. None of the participants from the compensated group had been hospitalized in the past year or exposed to the GCD form. They felt that although having a GCD was important, their inclination was to focus on staying healthy:

It's always prudent to guard against the downside, deal with that, but then right away focus the vast majority of effort to on finding a solution so it doesn't come to that. (Compensated, ACP1)

Participants wanted to feel confident their clinicians were doing everything possible to treat their disease. Some worried that ACP implied an expectation of deterioration and thus an orientation to management of symptoms rather than cure. When asked if it would be upsetting to have a clinician bring up GCD, one participant explained:

No. It would not upset me. I just want the goal to be recovery and not just management of deterioration. I don't want there to be an expectation of deterioration. (Compensated, ACP1)

Participants acknowledged that ACP conversations are difficult but they preferred frank information. While some worried that such conversations could reduce hope for recovery, most preferred realistic and direct information so that they could start making decisions:

I want to know everything upfront. I don't want surprises. … I'm okay with that [information] instead of sitting and waiting and wondering what is going to be the outcome. You need to know what could happen with CPR and how you will function after treatments. It's important to understand what to expect with liver problems like the toxins [encephalopathy]. (Contraindication to Transplant, ACP17)

All participants expressed having a trusting relationship with clinicians who knew them and their medical condition as key factors in determining which clinician should initiate GCD conversations. For those who had experienced minimal complications, this was most often a family physician, and for participants with more advanced cirrhosis, it was their cirrhosis clinician. Many expressed feeling uncomfortable discussing GCD with a care provider they did not know in an acute care setting:

We're not all going to be the same. To know your patient, you know their personality. You know what they can take. Whereas talking to somebody you don't really know all that well, you don't know what to expect. (Low SES, ACP6)

They explained that conversations with unfamiliar care providers could lead to lack of clarity:

I was in the hospital, in and out over two weeks at the holidays. One doctor would say this, one doctor would say that, nobody could tell me a straight answer, and I was like, to hell with that, I'm not doing this anymore. (Low SES, ACP4)

Predominantly, participants acknowledged their providers' skills in difficult conversations about illness trajectories and prognosis:

They are very comfortable talking to me and I am very comfortable talking to them. Yes, it is hard sometimes for doctors to come out and say things, but I hope they won't feel that way. (Medical Contraindication to Transplant, ACP17)

Participants on the transplant wait list described receiving information about risk of death on the wait list, yet none recalled discussions regarding EOL options. The focus on receiving a transplant meant that they received limited information with which to consider GCD decisions if they were no longer able to receive a transplant:

I think all of us have those ideas going through our heads, but that doesn't mean we have the right information to make that decision. (Transplant Wait Listed, ACP8)

Most participants described wanting examples about treatment options and consequences rather than statistics. They expressed a desire for the GCD explanations to be made in a stepwise manner. Those who had experienced decompensation wanted more specific information, such as illness trajectory graphs, examples as they pertained to quality of life, and options regarding EOL care. For those who had not experienced decompensation, there was a preference for the information to be provided simultaneously with recommendations about maintaining or improving health.

### 3.4. Perceptions of ACP/GCD Resources

Participants were invited to provide feedback on the ACP/GCD resources available. Many found the generalized videos informative and helpful. All participants had recommendations for customizing the videos specific to those with cirrhosis. Participants suggested a need for new resources to help them talk about EOL with their families. Although a medical order, the GCD is meant to be easily understood. All participants commented that the language on the GCD form was too complex for them to understand:

I don't know if it's just me being a bit thick, but some of this [GCD form] seems too technical, and if you look at the back it doesn't help. [note: the back of the form has descriptions of each GCD level]. (Transplant Wait Listed, ACP9)

## 4. Discussion

The aim of this study was to explore perceptions on ACP/GCD processes as they pertained to outpatients with cirrhosis. Lack of illness trajectory knowledge was a major barrier to ACP understanding. Limitations in patients' knowledge of cirrhosis progression and outcomes has been reported [16, 27], consistent with the findings in our study. GCD decisions at the time of hospitalization were common (n = 13/13); however, understanding the decisions or purpose of the GCD form upon recovery was sparse. Patient decision aids can help people prepare for ACP discussions. Despite being available online, there is no specific local process for providing patients with these resources, nor are the resources cirrhosis-specific. In patients with advanced cancers, decision aids are effective; however, authors likewise noted there was no process in place to standardize the use of these aids [28].

Nearly all participants mentioned involvement of family in the ACP process. They often expressed more concern over how family would cope versus themselves. In a recent review of the family experience in critical illness, many patients felt that they needed more information and that the information presented to them was confusing [29]. They also named family centered care as one of the most valuable aspects of ACP [30], validating the concerns of participants in our study. Conversely, participants who did not have an alternate decision maker expressed concern surrounding the impact this may have at EOL. Lack of alternate decision makers can present a challenge to clinicians caring for this population [31, 32] and early ACP discussions may be particularly important so there is clear understanding of the patient's wishes before complications develop [33].

Early anticipatory planning requires discussions be initiated in primary and outpatient care contexts [16]; however, there is currently no consensus on the most appropriate timing in cirrhosis. Hepatic encephalopathy is one of the most common reasons for cirrhosis hospitalization [34]. Thus, it is unsurprising that several participants listed encephalopathy as a barrier to being able to understand or remember their GCD.

For compensated participants, there was a general belief in the importance of ACP, but preference for care to be focused on improving function and providing self-health recommendations. Participants on the liver transplant list felt the focus of care was on receiving a transplant and not EOL options, even though they were aware of the significant risk of death while waiting for transplant. This dichotomy of planning for EOL while maintaining hope for a transplant is a barrier to ACP in several organ transplant candidate groups [9, 17, 35, 36].

Additionally, participants consistently preferred to have ACP discussions with a clinician who was known to them compared to an unknown hospital clinician. The importance of clinicians in providing direct, disease specific ACP information has been described in a lung disease population and our findings support this to be valid in cirrhosis [37].

To our knowledge, this is the first qualitative study examining ACP perceptions of participants at various stages of cirrhosis, allowing for analysis of experiences throughout disease trajectory. Therefore, our goal was information power [38] rather than analytic saturation for this specific topic. Individual interviews facilitated in depth identification of experiences and issues, whereas focus group participant interactions were rich and clearly elucidated where there was consensus and diversity on key concerns.

Strengths included the use of open-ended questions and encouragement of participants to raise their own questions, which was important to ensure the issues of importance to participants were elucidated. The dual role of the PI as both interviewer and clinical NP proved to be a strength, as those participants who knew her were comfortable and felt freer to express their opinions about their ACP experiences. Conversely, it is possible that participants who did not know the interviewer may have felt less comfortable expressing opinions. Several other potential limitations were noted. First, we recruited patients from a single locality and although our findings are similar to evaluations of ACP/GCD in Alberta patient populations [21], this remains a limitation. Second, there were limitations in sample size and diversity of the population. More than half of the participants (n = 9/17) had alcohol induced liver disease, a small portion (n = 3/17) were female, and none were less than 40 or more than 79 years of age. Although this can be considered fairly representative of a cirrhosis population, topics and issues specific to cirrhosis minorities, such as patients with less common cirrhosis etiologies, may not have be captured and limit the generalizability of our data. Finally, the use of focus groups provided the opportunity for interaction among participants; however, individual comfort with providing opposing opinions in a group setting may have limited the responses of some participants.

## 5. Conclusion

ACP conversations and GCD decisions in acute care situations may not be optimized for patients with cirrhosis and their families. These findings support the need for earlier ACP in the stable outpatient setting. Cirrhosis and primary care clinicians may play a key role in facilitating these discussions in their clinics, involving alternate decision makers, educating patients using high quality resources, and understanding their preferences.

## Figures and Tables

**Box 1:**
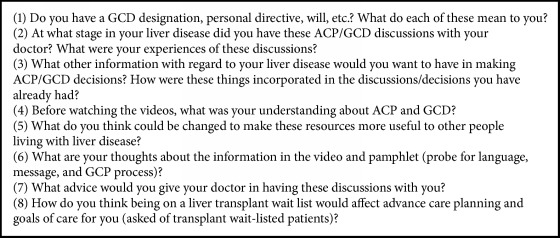
Participant demographic characteristics.

**Table 1:** Goals of Care Designation Levels.

**Goals of Care Designation**		**Chest Compressions**	**Intubation**	**ICU**	**Surgery**	**Site Transfer**	**Symptom Control**
Resuscitative Care	1	✓	✓	✓	✓	✓	✓
	2	X	✓	✓	✓	✓	✓
	3	X	X	✓	✓	✓	✓
Medical Care	1	X	X	X	✓	✓	✓
	2	X	X	X	Can consider, if required for symptom control		✓
Comfort Care	1	X	X	X			✓
	2	X	X	X	X	X	✓

From: AHS (2016) Goals of Care Designation (GCD) Pocket Card. Covenant Health Palliative Institute, August 24, 2016. Available at www.albertahealthservices.ca.

**Table 2:** Participant demographic characteristics.

**Demographics**	**Participants (n = 17)**
**Gender**	
Male	14
Female	3

**Age**	
40-49	2
50-59	6
60-69	8
70-79	1

**Etiology**	
Autoimmune Hepatitis	1
Non-alcoholic	4
Alcohol	8
Hepatitis C	2
Alcohol & Hepatitis C	1
Cryptogenic	1

**Table 3:** Participants' clinical status.

**Clinical status**	**Number** (n = 17)	**MELD Score** Mean (range)	**Child- Pugh Class** (Score)
Compensated	4	8.75 (8–10)	A (5): n= 4

Prior decompensation, not currently listed for transplant	3	13.6 (7-22)	A (5-6): n =2 C (11): n =1

Transplant Listed	3	15.3 (7-20)	A (6): n=1 B (9): n =1 C (10): n=1

Contraindication to Transplant	3	11 (8-17)	B (7-8): n =2 C (10): n =1

Low Socio-economic status	4	12.25 (9-18)	B (7-9): n=2 C (10-11): n=2

## Data Availability

Requests for data that support the findings of this study will be considered on request from the corresponding author (MC). The data is not publicly available due to it containing information that could compromise participant privacy/consent.
